# Supplementary material for: Comparative Transcriptome Analysis Provides Novel Insights into the Effect of Lipid Metabolism on Laying of Geese
Source: Animals (Basel). 2022 Jul 11;12(14):1775. doi: 10.3390/ani12141775 (PMC9311715; doi:10.3390/ani12141775)
Supplement: Supplementary file 1 [file animals-12-01775-s001.zip › animals-1797216-supplementary/Supplementary Table S2.pdf]

Supplementary Table S2. KEGG pathways enriched by DEGs of different groups

Group	KEGG_Name	Rich_factor	*P*_value	Input
Low.AF vs High.AF	AGE-RAGE signaling pathway in diabetic complications	0.040	0.000	*IL1B, NOS3, IL8, PIM1*
	NF-kappa B signaling pathway	0.040	0.000	*CCL19, IL1B, CCL4, IL8*
	Fluid shear stress and atherosclerosis	0.029	0.000	*NCF2, IL1B, NOS3, HMOX1*
	Malaria	0.061	0.000	*IL1B, CD36, IL8*
	Cytokine-cytokine receptor interaction	0.017	0.000	*CCL19, IL1B, BMP5, CCL4, IL8*
	Pathways in cancer	0.011	0.000	*IGF2, PIM1, HMOX1, KIT, FGF17, IL8*
	PI3K-Akt signaling pathway	0.014	0.000	*IGF2, DDIT4, NOS3, FGF17, KIT*
	Salmonella infection	0.036	0.000	*IL1B, CCL4, IL8*
	Hematopoietic cell lineage	0.031	0.000	*IL1B, CD36, KIT*
	Glycosaminoglycan degradation	0.105	0.000	*HYAL2, HPSE2*
	Viral protein interaction with cytokine and cytokine receptor	0.030	0.000	*CCL19, CCL4, IL8*
	Toll-like receptor signaling pathway	0.029	0.000	*IL1B, CCL4, IL8*
	MAPK signaling pathway	0.014	0.001	*IGF2, IL1B, FGF17, KIT*
	Phagosome	0.020	0.001	*NCF2, MARCO, CD36*
	Chemokine signaling pathway	0.016	0.002	*CCL19, CCL4, IL8*
	Legionellosis	0.036	0.003	*IL1B, IL8*
	Cytosolic DNA-sensing pathway	0.032	0.004	*IL1B, CCL4*
	Human cytomegalovirus infection	0.013	0.004	*IL1B, CCL4, IL8*
	Acute myeloid leukemia	0.030	0.004	*PIM1, KIT*
	Ras signaling pathway	0.013	0.004	*IGF2, FGF17, KIT*

Leishmaniasis	0.027	0.005	*NCF2, IL1B*
Pertussis	0.026	0.005	*IL1B, IL8*
Rheumatoid arthritis	0.022	0.007	*IL1B, IL8*
IL-17 signaling pathway	0.022	0.008	*IL1B, IL8*
TGF-beta signaling pathway	0.021	0.008	*BMP5, GREM1*
Amoebiasis	0.021	0.008	*IL1B, IL8*
MicroRNAs in cancer	0.010	0.008	*DDIT4, HMOX1, PIM1*
Chagas disease (American trypanosomiasis)	0.019	0.009	*IL1B, IL8*
Insulin resistance	0.019	0.010	*NOS3, CD36*
HIF-1 signaling pathway	0.018	0.010	*NOS3, HMOX1*
Neuroactive ligand-receptor interaction	0.009	0.012	*APEL, NPFF2, MC5R*
Yersinia infection	0.017	0.013	*IL1B, IL8*
Metabolic pathways	0.004	0.014	*NOS3, AMPD3, HYAL2, HMOX1, GADL1, HPSE2*
Osteoclast differentiation	0.016	0.014	*NCF2, IL1B*
Apelin signaling pathway	0.015	0.016	*NOS3, APEL*
Taurine and hypotaurine metabolism	0.091	0.016	*GADL1*
Breast cancer	0.014	0.018	*FGF17, KIT*
Phospholipase D signaling pathway	0.014	0.018	*IL8, KIT*
Non-alcoholic fatty liver disease (NAFLD)	0.013	0.018	*IL1B, IL8*
Influenza A	0.012	0.023	*IL1B, IL8*
Hepatocellular carcinoma	0.012	0.023	*IGF2, HMOX1*
NOD-like receptor signaling pathway	0.011	0.026	*IL1B, IL8*
Pantothenate and CoA biosynthesis	0.053	0.027	*GADL1*
Arginine biosynthesis	0.048	0.030	*NOS3*
Proteoglycans in cancer	0.010	0.032	*IGF2, HPSE2*
Rap1 signaling pathway	0.010	0.035	*FGF17, KIT*

	Antifolate resistance	0.032	0.043	*IL1B*
	beta-Alanine metabolism	0.030	0.046	*GADL1*
	Prion diseases	0.029	0.048	*IL1B*
	Metabolic pathways	0.010	0.000	*ECHA, GPX2, DGAT2, KBL, ADCY3, KMO, LIPH, HAOX2, QCR10, HAOX1, GYS2, QCR7, HGD, ALDOB, DGKG*
	Cholesterol metabolism	0.080	0.000	*LIPH, PCSK9, APOB, APOA2*
	Complement and coagulation cascades	0.051	0.000	*MBL, KNG1, MASP1, A1AT*
	Peroxisome	0.048	0.000	*HAOX1, HAOX2, PEX5, ALS*
	Fat digestion and absorption	0.073	0.000	*FABPL, DGAT2, APOB*
	Carbon metabolism	0.034	0.000	*HAOX1, ECHA, ALDOB, HAOX2*
	Glycerolipid metabolism	0.049	0.001	*LIPH, DGAT2, DGKG*
	Longevity regulating pathway - multiple species	0.048	0.001	*FOXA2, ALS, ADCY3*
No.AF vs Low.AF	Bile secretion	0.042	0.001	*NR0B2, OSTA, ADCY3*
	Huntington disease	0.021	0.002	*CYC, QCR10, QCR7, ALS*
	Vitamin digestion and absorption	0.083	0.002	*APOB, IF*
	Viral protein interaction with cytokine and cytokine receptor	0.030	0.003	*CCL19, CSF1R, CCL4*
	Glyoxylate and dicarboxylate metabolism	0.067	0.003	*HAOX1, HAOX2*
	Tryptophan metabolism	0.048	0.006	*KMO, ECHA*
	Parkinson disease	0.021	0.007	*CYC, QCR10, QCR7*
	Non-alcoholic fatty liver disease (NAFLD)	0.020	0.008	*CYC, QCR10, QCR7*
	Amyotrophic lateral sclerosis (ALS)	0.039	0.009	*CYC, ALS*
	cGMP-PKG signaling pathway	0.018	0.011	*KNG1, GAR1, ADCY3*
	Alzheimer disease	0.018	0.012	*CYC, QCR10, QCR7*
	Pathways in cancer	0.009	0.016	*CYC, CSF1R, KNG1, MMP2, ADCY3*

	Staphylococcus aureus infection	0.029	0.016	*MBL, MASP1*
	Chemokine signaling pathway	0.016	0.016	*CCL19, CCL4, ADCY3*
	p53 signaling pathway	0.028	0.017	*CYC, SESN2*
	Thyroid hormone synthesis	0.027	0.018	*GPX2, ADCY3*
	PPAR signaling pathway	0.026	0.019	*FABPL, APOA2*
	Cardiac muscle contraction	0.023	0.024	*QCR10, QCR7*
	Human cytomegalovirus infection	0.013	0.025	*CYC, CCL4, ADCY3*
	Longevity regulating pathway	0.022	0.026	*SESN2, ADCY3*
	Thermogenesis	0.013	0.026	*QCR10, QCR7, ADCY3*
	GnRH signaling pathway	0.022	0.028	*MMP2, ADCY3*
	TGF-beta signaling pathway	0.021	0.028	*THSD4, LTBP1*
	Endocrine resistance	0.020	0.031	*MMP2, ADCY3*
	Inflammatory mediator regulation of TRP channels	0.020	0.032	*KNG1, ADCY3*
	NF-kappa B signaling pathway	0.020	0.032	*CCL19, CCL4*
	Leukocyte transendothelial migration	0.018	0.039	*MMP2, JAM1*
	Cytokine-cytokine receptor interaction	0.010	0.048	*CCL19, CSF1R, CCL4*
No.AF vs High.AF	Cholesterol metabolism	0.140	0.000	*LIPC, APOB, ABCG5, ABCG8, LIPH, CD36, APOA2*
	Metabolic pathways	0.019	0.000	*GAMT, AMPD3, HAOX2, P4HA3, GADL1, RGN, CERS4, KYNU, KBL, DHRS9, DGAT2, KMO, SERA, TCB, LIPC, GPAT4, AADAT, LIPH, ADA2, GYS2, PLB1, ALDOB, ADCY2, G6PC, ACSL5, RDH11, EKI2*
	Fat digestion and absorption	0.146	0.000	*APOB, ABCG5, ABCG8, MTP, DGAT2, CD36*
	Complement and coagulation cascades	0.089	0.000	*MBL, F13A, KNG1, EPCR, TFPI1, A1AT, VWF*
	Hematopoietic cell lineage	0.072	0.000	*TFR1, CD34, CD36, KIT, CD2, IL1B, CD9*
	Bile secretion	0.083	0.000	*ABCG5, ADCY2, ABCG8, NR0B2, OSTA, NR1H4*

PI3K-Akt signaling pathway	0.031	0.000	*FOXO3, DDIT4, SGK2, COMP, G6PC, KIT, TLR2, GYS2, VWF, LPAR1, FGFR2*
Malaria	0.102	0.000	*TLR2, COMP, IL1B, CD36, IL8*
Pathways in cancer	0.021	0.000	*KNG1, ADCY2, PIM1, WNT2, RXRG, MMP2, KIT, RARB, LPAR1, FGFR2, IL8*
Glycerolipid metabolism	0.066	0.001	*LIPH, LIPC, GPAT4, DGAT2*
Longevity regulating pathway - multiple species	0.065	0.001	*FOXO3, FOXA2, ALS, ADCY2*
Carbon metabolism	0.043	0.001	*HAOX2, TCB, RGN, ALDOB, SERA*
Adipocytokine signaling pathway	0.058	0.001	*RXRG, G6PC, CD36, ACSL5*
PPAR signaling pathway	0.053	0.001	*RXRG, APOA2, CD36, ACSL5*
Salmonella infection	0.048	0.002	*IL1B, RHOG, CCL4, IL8*
Cytokine-cytokine receptor interaction	0.024	0.002	*CCL4, IL16, INHBC, CCR7, GDF15, IL1B, IL8*
Glycine, serine and threonine metabolism	0.075	0.002	*GAMT, KBL, SERA*
Phagosome	0.033	0.002	*TLR2, COMP, MBL, CD36, TFR1*
Tryptophan metabolism	0.071	0.002	*KMO, KYNU, AADAT*
ABC transporters	0.067	0.003	*ABCG8, ABCG5, ABCC9*
AGE-RAGE signaling pathway in diabetic complications	0.040	0.003	*IL1B, MMP2, IL8, PIM1*
NF-kappa B signaling pathway	0.040	0.003	*IL1B, CCL4, IL8, ZAP70*
Chagas disease (American trypanosomiasis)	0.039	0.003	*TLR2, IL1B, KNG1, IL8*
Toll-like receptor signaling pathway	0.038	0.003	*TLR2, IL1B, CCL4, IL8*
Glucagon signaling pathway	0.038	0.004	*TCB, GYS2, G6PC, ADCY2*
Th17 cell differentiation	0.037	0.004	*RORG, IL1B, RXRG, ZAP70*
Legionellosis	0.055	0.004	*TLR2, IL1B, IL8*
Chemokine signaling pathway	0.026	0.005	*FOXO3, ADCY2, CCL4, IL8, CCR7*

AMPK signaling pathway	0.033	0.005	*FOXO3, GYS2, G6PC, CD36*
Pantothenate and CoA biosynthesis	0.105	0.006	*GADL1, VNN1*
Proteoglycans in cancer	0.025	0.007	*TLR2, WNT2, IHH, MMP2, ANK1*
Inflammatory bowel disease (IBD)	0.046	0.007	*TLR2, RORG, IL1B*
Non-small cell lung cancer	0.045	0.007	*FOXO3, RARB, RXRG*
Purine metabolism	0.031	0.007	*AMPD3, ADA2, TCB, ADCY2*
Glycolysis / Gluconeogenesis	0.044	0.008	*TCB, ALDOB, G6PC*
Central carbon metabolism in cancer	0.043	0.008	*TCB, FGFR2, KIT*
Vitamin digestion and absorption	0.083	0.009	*APOB, PLB1*
Biosynthesis of amino acids	0.040	0.010	*ALDOB, TCB, SERA*
Phospholipase D signaling pathway	0.027	0.011	*LPAR1, ADCY2, IL8, KIT*
Gastric cancer	0.027	0.011	*RARB, WNT2, RXRG, FGFR2*
EGFR tyrosine kinase inhibitor resistance	0.038	0.011	*FOXO3, NRG2, FGFR2*
Peroxisome	0.036	0.013	*HAOX2, ALS, ACSL5*
Pentose phosphate pathway	0.067	0.014	*ALDOB, RGN*
ECM-receptor interaction	0.035	0.014	*COMP, VWF, CD36*
Antifolate resistance	0.065	0.015	*IL1B, FOLR1*
Circadian rhythm	0.065	0.015	*RORG, RORB*
Longevity regulating pathway	0.034	0.015	*FOXO3, SESN2, ADCY2*
Rheumatoid arthritis	0.033	0.016	*TLR2, IL1B, IL8*
TGF-beta signaling pathway	0.032	0.018	*THSD4, INHBC, FBN1*
Amoebiasis	0.032	0.018	*TLR2, IL1B, IL8*
Prion diseases	0.057	0.018	*IL1B, ALS*
Glycerophospholipid metabolism	0.031	0.019	*EKI2, GPAT4, PLB1*
Starch and sucrose metabolism	0.056	0.019	*GYS2, G6PC*
Primary immunodeficiency	0.054	0.020	*C2TA, ZAP70*

	African trypanosomiasis	0.054	0.020	*IL1B, KNG1*
	Inflammatory mediator regulation of TRP channels	0.030	0.021	*IL1B, KNG1, ADCY2*
	Viral protein interaction with cytokine and cytokine receptor	0.030	0.021	*CCL4, IL8, CCR7*
	Melanogenesis	0.030	0.021	*WNT2, ADCY2, KIT*
	Ferroptosis	0.050	0.023	*TFR1, ACSL5*
	Bladder cancer	0.049	0.024	*MMP2, IL8*
	Insulin resistance	0.028	0.025	*GYS2, G6PC, CD36*
	Ether lipid metabolism	0.043	0.031	*ENPP6, PLB1*
	Yersinia infection	0.025	0.033	*IL1B, ZAP70, IL8*
	Rap1 signaling pathway	0.019	0.033	*LPAR1, FGFR2, ADCY2, KIT*
	Arginine and proline metabolism	0.040	0.035	*P4HA3, GAMT*
	Relaxin signaling pathway	0.023	0.040	*RXFP1, MMP2, ADCY2*
	Human cytomegalovirus infection	0.018	0.041	*IL1B, CCL4, IL8, ADCY2*
	FoxO signaling pathway	0.023	0.041	*FOXO3, G6PC, SGK2*
	Human papillomavirus infection	0.015	0.042	*WNT2, VWF, TCB, MFNG, COMP*
	Insulin signaling pathway	0.022	0.045	*G6PC, GYS2, SH2B2*
	Estrogen signaling pathway	0.022	0.046	*MMP2, KCNJ6, ADCY2*
	Signaling pathways regulating pluripotency of stem cells	0.021	0.047	*WNT2, INHBC, FGFR2*
Low.L vs High.L	Glycerolipid metabolism	0.049	0.000	*DGAT1, LPIN2, LPIN1*
	Signaling pathways regulating pluripotency of stem cells	0.021	0.000	*TBX3, FZD5, WNT9A*
	mTOR signaling pathway	0.020	0.000	*FZD5, WNT9A, LPIN1*
	Proteoglycans in cancer	0.015	0.001	*TLR2, FZD5, WNT9A*

Basal cell carcinoma	0.032	0.002	*FZD5, WNT9A*
Metabolic pathways	0.004	0.002	*AOC3, LPIN2, LPIN1, DGAT1, ACSF3, G6PC2*
Glycerophospholipid metabolism	0.021	0.004	*LPIN2, LPIN1*
Melanogenesis	0.020	0.005	*FZD5, WNT9A*
PI3K-Akt signaling pathway	0.008	0.005	*TLR2, FOXO3, G6PC2*
AMPK signaling pathway	0.017	0.006	*FOXO3, G6PC2*
FoxO signaling pathway	0.015	0.007	*FOXO3, G6PC2*
Breast cancer	0.014	0.009	*FZD5, WNT9A*
Gastric cancer	0.013	0.009	*FZD5, WNT9A*
Hippo signaling pathway	0.013	0.010	*FZD5, WNT9A*
Cushing syndrome	0.013	0.010	*FZD5, WNT9A*
Wnt signaling pathway	0.013	0.011	*FZD5, WNT9A*
Hepatocellular carcinoma	0.012	0.012	*FZD5, WNT9A*
Pathways in cancer	0.006	0.015	*FZD5, WNT9A, CXCR4*
Chemokine signaling pathway	0.011	0.015	*FOXO3, CXCR4*
Phenylalanine metabolism	0.059	0.017	*AOC3*
Fatty acid biosynthesis	0.056	0.018	*ACSF3*
Human immunodeficiency virus 1 infection	0.009	0.018	*TLR2, CXCR4*
Vitamin digestion and absorption	0.042	0.024	*RBP2*
Maturity onset diabetes of the young	0.038	0.026	*NR5A2*
Galactose metabolism	0.032	0.031	*G6PC2*
beta-Alanine metabolism	0.030	0.032	*AOC3*
Tyrosine metabolism	0.028	0.035	*AOC3*
Starch and sucrose metabolism	0.028	0.035	*G6PC2*
Glycine, serine and threonine metabolism	0.025	0.039	*AOC3*
Fat digestion and absorption	0.024	0.040	*DGAT1*

	Human papillomavirus infection	0.006	0.041	*FZD5, WNT9A*
	Carbohydrate digestion and absorption	0.023	0.043	*G6PC2*
	ABC transporters	0.022	0.044	*ABCA4*
	Valine, leucine and isoleucine degradation	0.021	0.046	*ACSF3*
	Intestinal immune network for IgA production	0.020	0.047	*CXCR4*
	Malaria	0.020	0.047	*TLR2*
	Tuberculosis	0.017	0.000	*MOT2, CR1, SPHK1*
	Circadian rhythm	0.065	0.000	*PER3, NPAS2*
	VEGF signaling pathway	0.034	0.000	*SPHK1, NOS3*
	Circadian entrainment	0.021	0.001	*PER3, RASD1*
	Insulin resistance	0.019	0.001	*PTPRF, NOS3*
	Sphingolipid signaling pathway	0.017	0.001	*SPHK1, NOS3*
	Apelin signaling pathway	0.015	0.001	*SPHK1, NOS3*
	Calcium signaling pathway	0.010	0.002	*SPHK1, NOS3*
	Synthesis and degradation of ketone bodies	0.100	0.004	*BDH*
No.L vs Low.L	Arginine biosynthesis	0.048	0.008	*NOS3*
	Butanoate metabolism	0.036	0.011	*BDH*
	Metabolic pathways	0.002	0.016	*SPHK1, NOS3, BDH*
	Sphingolipid metabolism	0.021	0.018	*SPHK1*
	Malaria	0.020	0.019	*CR1*
	Arginine and proline metabolism	0.020	0.019	*NOS3*
	Mineral absorption	0.019	0.020	*COPT1*
	Legionellosis	0.018	0.021	*CR1*
	Renal cell carcinoma	0.014	0.026	*PRCC*
	Adherens junction	0.014	0.028	*PTPRF*
	Platinum drug resistance	0.014	0.028	*COPT1*

	Leishmaniasis	0.014	0.028	*CR1*
	RNA degradation	0.013	0.030	*MOT2*
	Complement and coagulation cascades	0.013	0.030	*CR1*
	Fc gamma R-mediated phagocytosis	0.011	0.036	*SPHK1*
	Hematopoietic cell lineage	0.010	0.037	*CR1*
	AGE-RAGE signaling pathway in diabetic complications	0.010	0.038	*NOS3*
	HIF-1 signaling pathway	0.009	0.041	*NOS3*
	Platelet activation	0.008	0.047	*NOS3*
	Relaxin signaling pathway	0.008	0.049	*NOS3*
No.L vs High.L	Metabolic pathways	0.022	0.000	*AOC3, AOC2, HCD2, SPHK1, G6PC2, LPIN2, CGT, LPIN1, HKDC1, XDH, FAHD1, TKT, P5CS, NRK2, SUOX, RIFK, GALT, BDH, SCD5, ALG12, EKI1, ACOT1, ACYP1, GCSP, HEXD, TKFC, AZIN2, OPLA, CANT1, PDE4B, CHAC1, AGT2*
	TGF-beta signaling pathway	0.074	0.000	*BMP6, INHBC, THSD4, INHBA, CHRD, FST, MIS*
	Glycine, serine and threonine metabolism	0.100	0.000	*AOC3, AOC2, AGT2, GCSP*
	Cholesterol metabolism	0.080	0.000	*LRP2, APOA4, PLTP, LCAT*
	Glycerophospholipid metabolism	0.052	0.000	*PLPP5, LCAT, LPIN2, EKI1, LPIN1*
	Cytokine-cytokine receptor interaction	0.027	0.000	*BMP6, EDA, IL2RG, FAS, INHBA, INHBC, CXCR4, MIS*
	Glycerolipid metabolism	0.066	0.000	*PLPP5, LPIN2, TKFC, LPIN1*
	Maturity onset diabetes of the young	0.115	0.001	*HNF4G, FOXA2, NR5A2*
	Inflammatory bowel disease (IBD)	0.062	0.001	*TLR2, RORG, TLR5, IL2RG*
	Galactose metabolism	0.097	0.001	*GALT, HKDC1, G6PC2*
	Calcium signaling pathway	0.031	0.001	*TPC1, ERBB2, SPHK1, CXCR4, P2RX2, P2RX5*
	Pathways in cancer	0.019	0.001	*ERBB2, STAT2, IL2RG, FAS, TRXR1, GLI1, WNT9A, CXCR4, PML, FGF22*

PPAR signaling pathway	0.053	0.001	*SCD5, FABPI, PLTP, PLIN1*
Proteoglycans in cancer	0.030	0.001	*ERBB2, FAS, SDC4, TLR2, IHH, WNT9A*
Tyrosine metabolism	0.083	0.001	*FAHD1, AOC3, AOC2*
Signaling pathways regulating pluripotency of stem cells	0.036	0.001	*TBX3, KLF4, INHBC, WNT9A, INHBA*
Hedgehog signaling pathway	0.064	0.003	*LRP2, IHH, GLI1*
Legionellosis	0.055	0.004	*TLR2, CR1, TLR5*
Carbon metabolism	0.034	0.005	*HKDC1, TKT, TKFC, GCSP*
Phenylalanine metabolism	0.118	0.005	*AOC3, AOC2*
AMPK signaling pathway	0.033	0.005	*STK11, SCD5, ULK1, G6PC2*
Human immunodeficiency virus 1 infection	0.024	0.008	*TLR2, PAK5, FAS, CCNB3, CXCR4*
Measles	0.029	0.009	*TLR2, FAS, STAT2, IL2RG*
Platinum drug resistance	0.041	0.009	*ERBB2, FAS, COPT1*
Biosynthesis of unsaturated fatty acids	0.074	0.011	*SCD5, ACOT1*
mTOR signaling pathway	0.026	0.012	*STK11, ULK1, WNT9A, LPIN1*
Neuroactive ligand-receptor interaction	0.018	0.013	*NPY4R, H3, VIPR2, P2RX2, P2RX5, APEL*
Endocytosis	0.020	0.013	*PML, TRFR, IL2RG, GBF1, CXCR4*
Circadian rhythm	0.065	0.015	*RORG, NPAS2*
PI3K-Akt signaling pathway	0.017	0.016	*ERBB2, IL2RG, STK11, G6PC2, TLR2, FGF22*
Fructose and mannose metabolism	0.061	0.016	*HKDC1, TKFC*
Base excision repair	0.061	0.016	*NEIL1, OGG1*
beta-Alanine metabolism	0.061	0.016	*AOC3, AOC2*
Starch and sucrose metabolism	0.056	0.019	*HKDC1, G6PC2*
Progesterone-mediated oocyte maturation	0.030	0.020	*CCNB3, CPEB3, KIF22*
Tuberculosis	0.022	0.020	*TLR2, MRC1, SPHK1, CR1*
Axon guidance	0.022	0.021	*PAK5, SLIT1, FPS, CXCR4*

	Transcriptional misregulation in cancer	0.022	0.022	*PML, PRCC, KLF3, LIPO*
	Th17 cell differentiation	0.028	0.024	*RORG, IRF4, IL2RG*
	Fat digestion and absorption	0.049	0.024	*APOA4, FABPI*
	HIF-1 signaling pathway	0.028	0.025	*HKDC1, ERBB2, TRFR*
	Carbohydrate digestion and absorption	0.045	0.027	*HKDC1, G6PC2*
	Epstein-Barr virus infection	0.020	0.029	*TLR2, FAS, STAT2, SUH*
	Sphingolipid metabolism	0.043	0.031	*SPHK1, CGT*
	Notch signaling pathway	0.042	0.032	*LFNG, SUH*
	Amino sugar and nucleotide sugar metabolism	0.042	0.032	*GALT, HKDC1*
	Valine, leucine and isoleucine degradation	0.042	0.032	*HCD2, AGT2*
	Caffeine metabolism	0.200	0.033	*XDH*
	Neomycin, kanamycin and gentamicin biosynthesis	0.200	0.033	*HKDC1*
	Malaria	0.041	0.033	*TLR2, CR1*
	Arginine and proline metabolism	0.040	0.034	*P5CS, AZIN2*
	cAMP signaling pathway	0.019	0.035	*PDE4B, GLI1, VIPR2, MIS*
	Purine metabolism	0.023	0.039	*PDE4B, CANT1, XDH*
	FoxO signaling pathway	0.023	0.041	*CCNB3, STK11, G6PC2*
	Human papillomavirus infection	0.015	0.041	*FAS, LFNG, STAT2, WNT9A, SUH*
	Glutathione metabolism	0.036	0.042	*CHAC1, OPLA*
	Insulin signaling pathway	0.022	0.044	*HKDC1, SH2B2, G6PC2*
	Apelin signaling pathway	0.022	0.044	*SPHK1, APEL, PLIN1*
	Riboflavin metabolism	0.125	0.049	*RIFK*
Low.O vs High.O	Regulation of actin cytoskeleton	0.019	0.000	*DIAP3, PAK6, FGF17, FGF16*
	Melanoma	0.042	0.000	*FGF17, FGF16, E2F1*
	Breast cancer	0.020	0.000	*FGF17, FGF16, E2F1*

	Gastric cancer	0.020	0.000	*FGF17, FGF16, E2F1*
	Ras signaling pathway	0.013	0.000	*PAK6, FGF17, FGF16*
	Pathways in cancer	0.006	0.004	*FGF17, FGF16, E2F1*
	Rap1 signaling pathway	0.010	0.007	*FGF17, FGF16*
	MAPK signaling pathway	0.007	0.013	*FGF17, FGF16*
	PI3K-Akt signaling pathway	0.006	0.018	*FGF17, FGF16*
	Circadian rhythm	0.032	0.019	*RORB*
	Bladder cancer	0.024	0.024	*E2F1*
	ABC transporters	0.022	0.027	*ABCC9*
	Ovarian steroidogenesis	0.020	0.029	*BMP15*
	Mitophagy - animal	0.015	0.038	*E2F1*
	Non-small cell lung cancer	0.015	0.039	*E2F1*
	Renal cell carcinoma	0.014	0.040	*PAK6*
	Adherens junction	0.014	0.042	*SNAI2*
	Pancreatic cancer	0.013	0.044	*E2F1*
	Glioma	0.013	0.044	*E2F1*
	Chronic myeloid leukemia	0.013	0.044	*E2F1*
	ErbB signaling pathway	0.012	0.049	*PAK6*
	Cardiac muscle contraction	0.012	0.050	*QCR7*
	ECM-receptor interaction	0.012	0.050	*SV2C*
No.O vs High.O	Rap1 signaling pathway	0.024	0.000	*EFNA3, RHOA, ADCY8, FGF17, FGF16*
	cAMP signaling pathway	0.023	0.000	*PTC1, RHOA, GRIA1, ADCY8, GAR1*
	Regulation of actin cytoskeleton	0.023	0.000	*DIAP3, FGF17, PAK6, RHOA, FGF16*
	Ras signaling pathway	0.022	0.000	*RHOA, EFNA3, PAK6, FGF17, FGF16*
	Pathways in cancer	0.011	0.000	*E2F1, ADCY8, PTC1, RHOA, FGF17, FGF16*
	Axon guidance	0.022	0.000	*PTC1, PAK6, EFNA3, RHOA*

Melanoma	0.042	0.000	*FGF17, FGF16, E2F1*
Human cytomegalovirus infection	0.018	0.000	*CCL2, RHOA, ADCY8, E2F1*
Breast cancer	0.020	0.002	*FGF17, FGF16, E2F1*
Gastric cancer	0.020	0.002	*FGF17, FGF16, E2F1*
cGMP-PKG signaling pathway	0.018	0.002	*RHOA, ADCY8, GAR1*
Ovarian steroidogenesis	0.041	0.003	*ADCY8, BMP15*
Chemokine signaling pathway	0.016	0.004	*CCL2, RHOA, ADCY8*
Human T-cell leukemia virus 1 infection	0.014	0.005	*CDS1, ADCY8, E2F1*
Long-term potentiation	0.030	0.005	*GRIA1, ADCY8*
p53 signaling pathway	0.028	0.006	*CDS1, SESN2*
ECM-receptor interaction	0.023	0.009	*AGRIN, SV2C*
Longevity regulating pathway	0.022	0.009	*ADCY8, SESN2*
Circadian entrainment	0.021	0.011	*GRIA1, ADCY8*
Pancreatic secretion	0.020	0.011	*RHOA, ADCY8*
Endocrine resistance	0.020	0.011	*ADCY8, E2F1*
MAPK signaling pathway	0.010	0.012	*EFNA3, FGF17, FGF16*
T cell receptor signaling pathway	0.019	0.012	*PAK6, RHOA*
MicroRNAs in cancer	0.010	0.012	*RHOA, EFNA3, E2F1*
Parathyroid hormone synthesis, secretion and action	0.019	0.013	*RHOA, ADCY8*
Glutamatergic synapse	0.018	0.015	*GRIA1, ADCY8*
Yersinia infection	0.017	0.016	*CCL2, RHOA*
Cell cycle	0.016	0.017	*CDS1, E2F1*
Platelet activation	0.016	0.017	*RHOA, ADCY8*
PI3K-Akt signaling pathway	0.008	0.019	*EFNA3, FGF17, FGF16*
Vascular smooth muscle contraction	0.015	0.019	*RHOA, ADCY8*

Fluid shear stress and atherosclerosis	0.014	0.021	*CCL2, RHOA*
Phospholipase D signaling pathway	0.014	0.024	*RHOA, ADCY8*
Retrograde endocannabinoid signaling	0.014	0.024	*GRIA1, ADCY8*
mTOR signaling pathway	0.013	0.025	*RHOA, SESN2*
Oxytocin signaling pathway	0.013	0.025	*RHOA, ADCY8*
Cushing syndrome	0.013	0.026	*ADCY8, E2F1*
Cellular senescence	0.013	0.027	*CDS1, E2F1*
Jak-STAT signaling pathway	0.012	0.028	*GFAP, CISH*
NOD-like receptor signaling pathway	0.011	0.033	*CCL2, RHOA*
Calcium signaling pathway	0.010	0.038	*ADCY8, P2RX5*
Vitamin digestion and absorption	0.042	0.039	*APOA4*
Focal adhesion	0.010	0.040	*PAK6, RHOA*
Epstein-Barr virus infection	0.010	0.041	*CR2, E2F1*
Proteoglycans in cancer	0.010	0.042	*PTC1, RHOA*
Phototransduction	0.036	0.045	*GAR1*
