# Supplementary material for: Comparative Transcriptome Analysis Provides Novel Insights into the Effect of Lipid Metabolism on Laying of Geese
Source: Animals (Basel). 2022 Jul 11;12(14):1775. doi: 10.3390/ani12141775 (PMC9311715; doi:10.3390/ani12141775)
Supplement: Supplementary file 1 [file animals-12-01775-s001.zip › animals-1797216-supplementary/Supplymentary Table S1.pdf]

Supplementary Table S1. Basic information of mRNA sequencing data of all samples in this study

Sample	Raw Reads	Clean Reads	Q20(%)	Q30(%)	GC Content(%)	Mapping rate(%)
D-F-1	21,384,849	20,473,606	97.44	92.98	52.62	90.07
D-F-2	25,965,237	24,830,497	97.48	93.05	51.95	90.40
D-F-3	20,395,510	19,448,506	97.55	93.22	52.66	90.01
D-G-1	22,813,103	21,840,834	97.7	93.4	50.66	92.64
D-G-2	24,724,057	23,652,750	97.72	93.44	50.48	92.54
D-G-3	26,265,682	25,119,718	97.73	93.44	50.53	92.67
D-L-1	23,708,555	22,632,005	97.29	92.69	53.84	89.47
D-L-2	31,052,061	29,064,020	97.59	93.53	52.49	90.05
D-L-3	21,568,074	20,278,438	97.52	93.27	54.56	88.53
G-F-1	27,261,703	25,933,684	97.34	92.66	51.47	90.05
G-F-3	23,974,555	22,748,942	97.52	93.14	51.34	90.34
G-F-5	27,712,269	26,696,933	97.37	93.03	51.78	90.04
G-G-1	23,990,273	21,797,518	97.98	93.83	46.02	93.09
G-G-3	22,992,233	20,929,390	98.01	93.89	46.36	93.44
G-G-5	21,905,543	20,754,905	97.61	93.21	50.33	91.92
G-L-1	21,769,900	20,793,578	97.5	93.06	51.73	90.25
G-L-3	23,236,466	22,169,320	97.56	93.2	51.57	90.37
G-L-5	21,172,059	20,114,623	97.56	93.25	51.8	90.37
W-F-1	23,900,638	22,817,637	97.64	93.45	53.16	90.23
W-F-4	22,219,622	21,407,675	97.65	93.36	53.03	90.35
W-F-5	24,135,438	23,148,683	96.91	91.55	51.28	91.09
W-G-1	44,085,543	42,766,974	97.67	93.78	55.2	86.26
W-G-4	26,989,276	25,844,345	97.53	93.23	51.67	91.39
W-G-5	21,990,247	20,942,201	97.81	93.7	51.88	91.77
W-L-1	21,666,563	20,977,514	98.09	95.01	53.72	90.56
W-L-4	24,835,258	23,944,442	97.88	94.63	53.89	89.27
W-L-5	30,550,464	29,378,115	98.07	94.98	52.72	90.16
